# Seed Protection of *Solanum lycopersicum* with *Pythium oligandrum* against *Alternaria brassicicola* and *Verticillium albo-atrum*

**DOI:** 10.3390/microorganisms10071348

**Published:** 2022-07-04

**Authors:** Kateřina Bělonožníková, Veronika Hýsková, Marie Vašková, Tomáš Křížek, Kateřina Čokrtová, Tomáš Vaněk, Lucie Halířová, Michal Chudý, Antoniana Žufić, Helena Ryšlavá

**Affiliations:** 1 Department of Biochemistry, Faculty of Science, Charles University, Hlavova 2030, 128 43 Prague 2, Czech Republic; katerina.belonoznikova@natur.cuni.cz (K.B.); veronika.hyskova@natur.cuni.cz (V.H.); marie.ouskova@natur.cuni.cz (M.V.); tomas.krizek@natur.cuni.cz (T.K.); katerina.cokrtova@seznam.cz (K.Č.); halirova.lucie@seznam.cz (L.H.); chudy.michal.k@gmail.com (M.C.); zuficnina@gmail.com (A.Ž.); 2 Department of Analytical Chemistry, Faculty of Science, Charles University, Hlavova 2030, 128 43 Prague 2, Czech Republic; 3 Biopreparáty, spol. s r.o., Tylišovská 1, 160 00 Prague 6, Czech Republic; vanek@biopreparaty.eu

**Keywords:** antioxidants, capillary electrophoresis, fungal diseases, plant protection, seed-coating

## Abstract

*Pythium oligandrum*, strain M1, is a soil oomycete successfully used as a biological control agent (BCA), protecting plants against fungal, yeast, and oomycete pathogens through mycoparasitism and elicitor-dependent plant priming. The not yet described *Pythium* strains, X42 and 00X48, have shown potential as BCAs given the high activity of their secreted proteases, endoglycosidases, and tryptamine. Here, *Solanum lycopersicum* L. cv. Micro-Tom seeds were coated with *Pythium* strains, and seedlings were exposed to fungal pathogens, either *Alternaria brassicicola* or *Verticillium albo-atrum*. The effects of both infection and seed-coating on plant metabolism were assessed by determining the activity and isoforms of antioxidant enzymes and endoglycosidases and the content of tryptamine, amino acids, and heat shock proteins. Dual culture competition testing and microscopy analysis confirmed mycoparasitism in all three *Pythium* strains. In turn, seed treatment significantly increased the total free amino acid content, changing their abundance in both non-infected and infected plants. In response to pathogens, plant Hsp70 and Hsp90 isoform levels also varied among *Pythium* strains, most likely as a strategy for priming the plant against infection. Overall, our results show in vitro mycoparasitism between *Pythium* strains and fungal pathogens and *in planta* involvement of heat shock proteins in priming.

## 1. Introduction

Fungal spores ubiquitously infect agriculturally important crops, especially contaminating cereals, nuts, fruits, and vegetables. Some fungal pathogens are so destructive that cultivation requires fungicides, which are potentially harmful for human health [1]. Among such pathogens, *Verticillium* spp. is responsible for wilt diseases that affect over 200 hosts. In particular, *Verticillium albo-atrum* infects the vascular system of many dicotyledonous plant species, and its fungal isolates secrete an arsenal of enzymes that degrade plant cell wall components, such as cellulose, pectin, hemicelluloses, and proteins, and secrete necrosis- and ethylene-inducing proteins, which play a key role in pathogenesis [2]. In turn, *A. brassicicola*, a necrotrophic fungal pathogen, stands out for producing AB mycotoxin (protein approximately 35 kDa), hydrolytic enzymes, and necrosis-related proteins on crops grown worldwide [3,4]. Accordingly, to avoid using fungicides, fungal pathogens, such as *A. brassicicola* and *V. albo-atrum*, must be prevented from destroying crops through biological control agents (BCAs).

Increasingly used as a BCA, *Pythium oligandrum*, Dreschler, is a soil oomycete that protects plants against fungi, yeasts, and oomycetes including very dangerous pathogens (e.g., *Fusarium oxysporum*, *Phytophthora parasitica*, *V. dahlie*, *Botrytis cinerea*, and *Aphanomyces cochlioides*, and even other *Pythium* species) [5,6,7]. At least three levels of action account for *P. oligandrum* efficacy in plant protection [5]. First, *P. oligandrum* acts as a mycoparasite, secreting various hydrolytic enzymes that degrade the host cell wall (especially chitinases, cellulases, endo-β-1,3-glucanases, various exoglycosidases, proteases, and phosphatases) and compete with pathogens for nutrients and space. As a case in point, the *P. oligandrum* genome contains 114 glycoside hydrolases, of which 79 are secreted [8]. However, when colonizing the plant root system, *P. oligandrum* penetrates through the apoplast without causing damage and only partly affects cell wall components, most likely due to the absence of the enzyme cutinase [6,8]. Second, *P. oligandrum* produces numerous elicitors termed oligandrins (i.e., OLI-D1, OLI-D2, and OLI-S1) and cell wall protein fractions (i.e., POD-1, POD-1a, POD-1b, POD-2, and POS-1) [5,9,10,11,12]. These low-molecular mass proteins induce plant defense systems, thus initiating both local- and systemic-induced resistance against fungal, oomycete, and bacterial pathogens [6]. Third, *P. oligandrum* contains other proteins that can affect the immune system of a plant [5]. Recently, 35 RXLR effector proteins have also been identified in the *P. oligandrum* genome, one of which induces plant defense responses [13]. Other proteins possibly involved in inducing defense reactions include necrosis- and ethylene-inducing peptide1-like proteins (NLPs), two of which activate defensin genes without causing the accumulation of reactive oxygen species [14]. In tomato plants, more specifically, *P. oligandrum*-mediated resistance involves the activation of ethylene (ET)- and jasmonic acid (JA)-dependent signaling pathways [15,16,17,18]. Finally, *P. oligandrum* supplements the plant root system with tryptamine, a precursor of the growth phytohormone auxin, thus enhancing plant fitness [19].

Heat shock protein (Hsp) functions range from protein folding and inhibition of aggregate formation to defense against plant stress. Hsps are classified according to sequence similarity, although they are still designated by molecular weight. Hsp70s are localized in the cytoplasm, mitochondria, chloroplasts, and endoplasmic reticulum [20]. In *Nicotiana tabacum*, this family has 61 members with molecular weights ranging from 16 to 100 kDa, individual members of which are expressed in response to phytohormones, high or low temperature, drought, wounding, or fungal pathogens [21]. Preliminary results of tomato plants also indicate that 23 candidate genes of the tomato Hsp70 family with molecular weights ranging from 21.3 to 98.8 kDa [22] and at least seven members of the Hsp90 tomato gene family may be involved in the response to stress, including resistance to *Tomato yellow leaf curl virus* [23]. Notwithstanding these advances in the identification of signaling pathways and elicitors by *P. oligandrum* in activating tomato plant resistance, the extent of protection depends on many other parameters including the application method, i.e., spraying, watering, or seed treatment [5].

In previous research, we have shown that seed-coating alone leads to a number of metabolic changes in rapeseed plants, including in free amino acids, phytohormones, and glucosinolates, with significant differences in enzyme secretion and elicitors among *P. oligandrum* strains [24]. However, the question as to whether tomato seed-coating with *P. oligandrum* protects against fungal pathogens, such as *V. albo-atrum* and *A. brassicicola*, remains unanswered. *Verticillium* spp. are soil-borne plant pathogens, but plant resistance against pathogens largely involves isolating the fungus in contained sections of xylem tissues and, subsequently, eliminating the fungus [25]. In turn, *A. brassicicola* causes more yield losses in Brassicaceae than in Resedaceae and Solanaceae (including tomato plants) for which this fungus is considered an occasional or weak pathogen [26].

In this study, we aimed to assess whether a seed treatment with *Pythium* strains affected the metabolism of model crop plant *Solanum lycopersicum* L. cv. Micro-Tom and induced defense responses against the fungal pathogens *A. brassicicola* and *V. albo-atrum*. For this purpose, we chose the most advantageous strains, based on the findings of our previous study, i.e., M1, 00X48, and X42 [24]. *P. oligandrum* M1 is a commercially available plant protection strain. *P. oligandrum* 00X48 has shown even higher cellulase, chitinase, endo-β-1,3-glucanase, serine protease activities, and concentrations of tryptamine secreted to the medium, thereby demonstrating its potential in both mycoparasitism and plant growth enhancement. Lastly, *Pythium* X42, a newly found (not yet described) *Pythium* species with the closest affinity to *Pythium* sp. E26 JN863988, has a unique protein profile and the highest protease activity, total phenolic content, and secretion of both tryptophan and tryptamine to the medium, which are positively correlated with auxin levels in host rapeseed leaves, i.e., support plant growth. Therefore, we hypothesized that the novel *Pythium*-strains X42 and 00X48 could be effective biological control agents against fungal diseases directly (i.e., act as mycoparasites) as well as indirectly by inducing plant priming and help to maintain plant fitness. Thus, *Pythium* strains were exposed to the fungal pathogens *A. brassicicola* and *V. albo-atrum* in vitro and *in planta* to determine whether these strains are suitable biological control agents by analyzing (i) interactions between *Pythium* strains and *A. brassicicola* and *V. albo-atrum* and (ii) the effects of seed-coating with *Pythium* strains on the metabolism of tomato plants exposed to these fungal pathogens.

## 2. Materials and Methods

### 2.1. In Vitro Dual Culture Tests of Pythium Strains against A. brassicicola/V. albo-atrum

Dual culture tests were performed on solid malt extract agar (MEA, Merck, Darmstadt, Germany) medium in triplicates. The small plugs (5 × 5 mm) of agar plate with cultures of plant pathogens *A. brassicicola* and *V. albo-atrum* were placed 15 mm from the edge of the test MEA plate. The phytopathogens were incubated for 72 h (dark, 24 °C, incubator Memmert IN75, Memmert, Schwabach, Germany) before adding the *Pythium* strains to the opposite side of the pathogen plug. The MEA plates were then incubated for 16 days (dark, 24 °C). The plates inoculated only with pathogens served as negative controls. Microscopic observation of the dual culture was performed after 24 h, i.e., a few hours after first contact between the hyphae of pathogenic fungi and *Pythium* strains (Intracomicro FL BMS, Prague, Czech Republic). After 15 days, the mycelial radial growth of the pathogen on a control plate (r_1_) and in the direction of the antagonistic *Pythium* strain (r_2_) was measured, and the percentage inhibition (I%) in pathogen growth was calculated according to the formula: I% = [(r_1_ − r_2_)/r_1_] × 100 [27]. The ability of each *Pythium* strain to parasitize the fungi was assessed based on the visible overgrowth of the pathogen colony, pathogen hyphae growth inhibition, and loss of or inability to change pigmentation. In addition, the ability of *Pythium* sp. hyphae to encircle pathogen hyphae at sites of early contact between the two colonies was also evaluated.

### 2.2. Plant Material

*S. lycopersicum* L. cv. Micro-Tom seeds were treated with lyophilized *Pythium* spp. inoculum (oomycete biomass with medium inoculum) containing 12.0 × 10^6^ oospores g^−1^. *Pythium* spp. inocula were prepared as in [24]. Seeds were inoculated in a rotating seed machine at a concentration of 5 g inoculum/kg seed. Each mixture was moistened with deionized water, exposed to continuous rotation for 5 min, and then left to dry and stored at room temperature. Both treated and untreated *S. lycopersicum* L. cv. Micro-Tom seeds were sown in pots with soil and grown for four weeks in growth chambers under 16/8 h light/dark period, ca. 150 μmol (photon) m^−2^ s^−1^ irradiance, at 20 °C, and 60% relative humidity. In the second week of growth, the plants were infected with *A. brassicicola* and *V. albo-atrum.* After two weeks of growth, samples of all leaves from each group were immediately frozen in liquid N_2_ and kept at −80 °C.

### 2.3. Detection and Quantification of A. brassicicola and V. albo-atrum by qRT-PCR

Total DNA was isolated from leaves according to the protocol of the DNeasy Plant Mini kit (Qiagen, Hilden, Germany) for pathogen DNA detection. Subsequent quantitative RT-PCR reactions were performed using SYBR Green chemistry (SsoAdvanced Universal SYBR Green Supermix, Bio-Rad, Hercules, CA, USA) under the following conditions: 50 °C—2 min; 98 °C—2.5 min; 40 cycles: 98 °C—10 s, 60 °C—30 s on a Bio-Rad CFX Connect (Bio-Rad, USA). Each reaction mixture contained 15 ng of DNA sample at the beginning. The primers for *A. brassicicola* were constructed based on its genome (F: 5-TCCGTAGGTGAACCTGC-3, R: 5-TCCTCCGCTTATTGATATGC-3). For *V. albo-atrum*, the standard fungal ITS region was used to design the primers (F: 5-TCCGTAGGTGAACCTGCG-3, R: 5-CGCTGCGTTCTTCATCG-3). Actin was used as a standard gene (F: 5-CCTCTCAACCCGAAAGCCAA-3, R: 5-CATCACCAGAGTCGAGCACA-3).

### 2.4. Protein Concentration

Protein content was measured spectrophotometrically at 595 and 450 nm using a protein assay solution (Bio-Rad, USA) with bovine serum albumin as a standard [28].

### 2.5. Determination of Free Amino Acids and Tryptamine

Free amino acids were determined according to [29] by capillary electrophoresis with a contactless conductivity detector to separate 20 proteinogenic amino acids in an acidic background electrolyte. All electrophoretic experiments were conducted in a fused-silica capillary (Polymicro Technologies, Phoenix, AZ, USA) using a G7100A Capillary Electrophoresis System (Agilent Technologies, Waldbronn, Germany) with a contactless conductivity detector. The detector consisted of two 4 mm long cylindrical electrodes, with a 1 mm insulation gap. The inner diameter of the electrodes was 400 μm.

### 2.6. Phenolics and Antioxidant Capacity

The total phenolic content was determined using the standard Folin–Ciocalteu colorimetric method. The ferric reducing antioxidant potential (FRAP) assay was used to determine the antioxidant capacity [24].

### 2.7. Enzyme Activity

Enzyme activity was determined spectrophotometrically (Helios α, Thermo-Spectronics, Pine Island, MN, USA) and expressed as activity in μmol of the respective product (substrate decreased) per min per g of fresh plant weight (F.W.). Frozen leaf samples (0.5 g) were ground in liquid N_2_ with 1% polyvinylpyrrolidone. For each enzyme activity assay, 1.5 mL of extraction buffer was added (i.e., 0.1 M Tris–HCl, pH 7.8, 1 mM EDTA, 10 mM DTT, and 5 mM MgCl_2_) to the ground tissue. For Western blotting, 0.5 mL of extraction buffer (i.e., 0.13 M Tris–HCl, pH 6.8, 20% (*w*/*v*) sucrose, 3% (*w*/*v*) SDS, 0.5% (*w*/*v*) 2-mercaptoethanol, and 0.05% (*w*/*v*) bromophenol blue) was added. The homogenate was centrifuged at 13,000× *g* for 15 min, and the resulting supernatant was used for measurements. Catalase (CAT, EC 1.11.1.6) activity was detected at 240 nm as the H_2_O_2_ decomposition rate. Malic enzyme (NADP-ME, EC 1.1.1.40), glucose-6-phosphate dehydrogenase (G6PDH, EC 1.1.1.49), shikimate dehydrogenase (SDH, EC 1.1.1.25), and glutathione reductase (GR, EC 1.8.1.7) activities were assayed as NADPH production or consumption at 340 nm. Glutathione-S-transferase (GST, EC 2.5.1.18), peroxidase (PXs, EC 1.11.1.7), ascorbate peroxidase (APOD, EC 1.11.1.11), and superoxide dismutase (SOD, EC 1.15.1.1) isozyme patterns and activities were assessed after separation by 10% native PAGE and specific detections in gel [24,30,31].

### 2.8. Immunochemical Methods

Cytoplasmic Hsp70 and Hsp90.1 isoforms were immunochemically detected using specific primary antibodies (AS08371 and AS08346, Agrisera, Vännäs, Sweden) on nitrocellulose membranes (0.45 μm; Schleicher & Schuell, Dassel, Germany), after protein transfer from a 12% gel following SDS electrophoresis [32], loading 10 µg protein, and visualized as described previously [33]. Secondary polyclonal goat antibody against rabbit antibody was conjugated with alkaline phosphatase (Sigma-Aldrich, St. Louis, MO, USA).

### 2.9. Statistical Analysis

Each plant group was represented by at least 10 plants. All measurements were performed in at least triplicates. Data were analyzed by one-way analysis of variance (ANOVA) using Holm–Sidak multiple comparison test. The mean of each column was compared with the mean of every other column. In addition, two-way ANOVA for comparing *Pythium*-treatment vs. fungal infection, and *t*-tests were performed. All differences were considered significant at *p* ≤ 0.05. Normality statistic was performed according to Shapiro–Wilk (sample size ≤ 5000). Statistics was calculated in SigmaPlot 12.5. Details about ANOVA results can be found in the Supplementary Materials.

## 3. Results

### 3.1. Interactions between Pythium Strains and Fungal Phytopathogens

**Figure 1:**
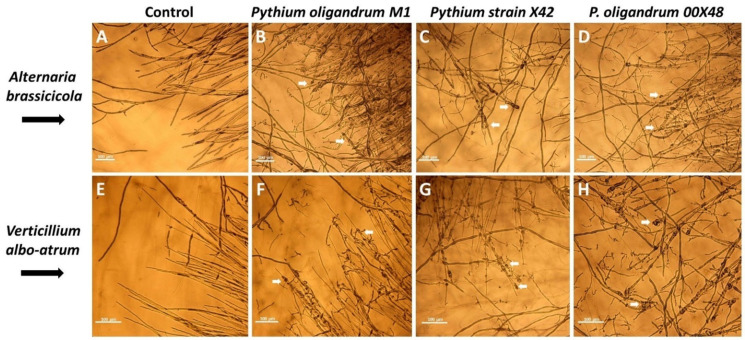
Interactions between *Pythium* strains and fungal pathogens *A. brassicicola* (**A**–**D**) and *V. albo-atrum* (**E**–**H**) after 24 h on a scale of 100 μm. Control growth of fungal pathogens (**A**,**E**); *P. oligandrum* M1 (**B**,**F**); *Pythium* X42 (**C**,**G**); *P. oligandrum* 00X48 (**D**,**H**). Arrows point at the sites where *Pythium* strains coil around the fungal hyphae.

*Pythium* strains were screened using in vitro dual culture assays for their ability to suppress *A. brassicicola* and *V. albo-atrum* mycelial growth by showing either mycoparasitism and/or antibiosis and/or competition for nutrients and space. *A. brassicicola* and *V. albo-atrum* were incubated for 72 h before inoculation with the *Pythium* strains. Twenty-four hours after co-inoculation, the first contact of both cultures, *Pythium* strain, and fungal pathogen, was established. Microscopic analysis revealed mycoparasitism hallmarks for all three *Pythium* strains because the hyphae of *V. albo-atrum* and *A. brassicicola* were both significantly coiled, displaying morphological changes (Figure 1).

**Figure 2:**
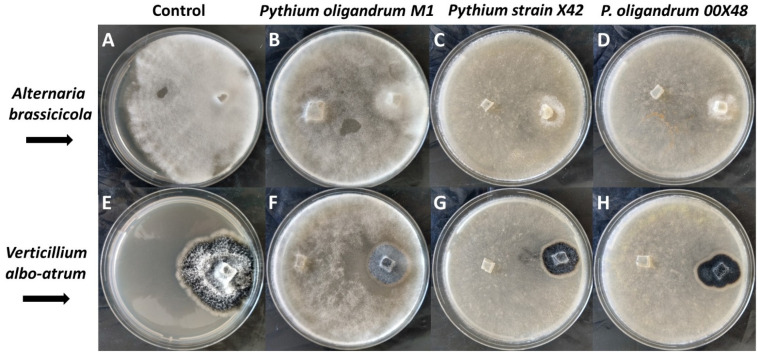
Dual culture plate competition tests between *Pythium* strains and fungal pathogens *A. brassicicola* (**A**–**D**) and *V. albo-atrum* (**E**–**H**). Control growth of fungal pathogens (**A**,**E**); *P. oligandrum* M1 (**B**,**F**); *Pythium* X42 (**C**,**G**); *P. oligandrum* 00X48 (**D**,**H**).

The inhibition percentage (I%) of each *Pythium* strain against both fungal pathogens was calculated from the dual culture assay (Figure 2). The *Pythium* strain X42 showed the highest inhibition percentage for both *V. albo-atrum* and *A. brassicicola*, 67.1 ± 2.0% ^a^ and 91.5 ± 1.1% ^B^, respectively, followed by both 00X48 (65.3 ± 2.7% ^a^ and 89.0 ± 0.0% ^A,B^, different letters in superscript mean a significant difference, for more see Table S1) and M1 (66.5 ± 3.1% ^a^ and 87.2 ± 1.8% ^A^) 16 days after inoculation. A statistically significant difference in inhibition percentage was found only between X42 and M1 against *A. brassicicola* (Table S1). The colonies of both pathogens were overgrown by *Pythium* strains 24 h after first contact, with no subsequent growth of pathogens colonies.

*Pythium* strains showed a rapid overgrowth when interacting with *A. brassicicola*. When interacting *V. albo-atrum*, *Pythium* strains first formed a contact inhibition zone of various sizes at the level of substrate mycelium, followed by the production of an aerial mycelium, which overgrew the fungal pathogen. *P. oligandrum* M1 produced most aerial hyphae. Thus, its overgrowth was more significant in contact with *V. albo-atrum* than with *A. brassicicola* colonies (Figure 2F).

### 3.2. Seed-Coated Tomato Plants with Pythium Strains Exposed to Fungal Pathogens

**Figure 3:**
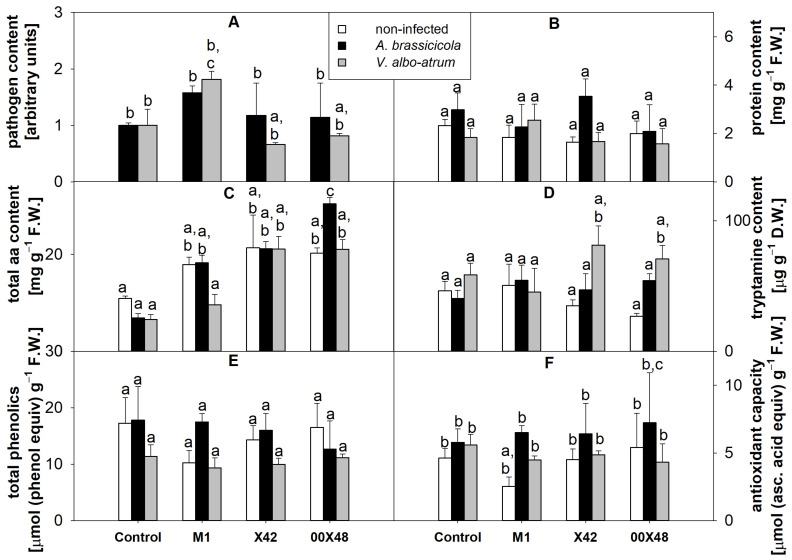
Relative content of *A. brassicicola* (black columns) and *V. albo-atrum* (grey columns) determined by RT-qPCR (**A**); content of total proteins (**B**); total free amino acids (**C**); tryptamine (**D**); total phenolics (**E**); antioxidant capacity (**F**) of tomato leaf extracts from plants grown from seeds treated with a *Pythium* strain (i.e., M1, X42, and 00X48) and from untreated control seeds (**Control**). Relative pathogen content (**A**) was expressed to the standard gene *actin*. Different letters above each bar denote significant differences (*p* ≤ 0.05) between plant groups according to one-way ANOVA (Holm–Sidak). The same letters above a bar indicate that no significant differences were found among the groups. Each column bar represents the mean ± SD. The degrees of freedom, F-value, and *p*-values can be found in Table S2. Abbreviations: asc. acid, ascorbic acid; D.W., dry weight; F.W., fresh weight.

*S. lycopersicum* L. (cv. Micro-Tom) seeds were treated with lyophilized *Pythium* strains and, after two weeks of growth, the plants were challenged with *A. brassicicola* and *V. albo-atrum* and grown for another two weeks. The pathogen content was confirmed by RT-qPCR in leaves of infected plants. Seed-coating did not cause significant differences in pathogen content in most groups. The highest number of fungal pathogens was detected in the M1 leaves, while 00X48 and X42 showed a lower *V. albo-atrum* content than the control (Figure 3A).

In general, seed-coating with *Pythium* strains (i.e., M1, X42, and 00X48) did not significantly change the protein content among the experimental groups (Figure 3B) but had a positive effect on the total free amino acid content of leaves from all treated groups in comparison with the control. This result was statistically confirmed by two-way ANOVA, with a Pearson coefficient of 0.018 (Table S3). The highest increase in the content of total free amino acids (2.8-fold of the non-infected control) was found in leaves of infected *A. brassicicola* plants treated with the strain 00X48 (Figure 3C). *Pythium* strains can supplement plants with tryptamine, a metabolite of the amino acid tryptophan and an auxin precursor. After infection with *V. albo-atrum*, the tryptamine content increased 2.3 and 2.7 times in plants treated with the strains X42 and 00X48, respectively (Figure 3D). The total phenolics (Figure 3E) and antioxidant capacity (Figure 3F) did not differ among most experimental groups.

In addition to the total free amino acid content, tomato seed-coating with *Pythium* strains also affected the representation of individual amino acids (Table 1). Of all studied strains, X42 increased the content of most individual amino acids in uninfected plants the most, especially methionine, glycine, and proline. The content of some amino acids decreased, both due to the *Pythium* treatment and infection. The decrease in glutamate content was significant, especially when treating the seeds with M1. The graphical representation of other amino acids did not show such a clear trend in all strains. For example, the methionine content increased more than five-fold upon seed-coating with X42 and two-fold with M1, but strain 00X48 decreased the content of this amino acid by half. Infection with fungal pathogens mainly led to a reduction in the content of a number of free amino acids and more markedly so upon infection with *A. brassicicola* than with *V. albo-atrum*. In contrast, both fungal pathogens increased the content of aspartate (Table 1).

### 3.3. Stress Conditions Affect Plant Metabolism

In response to biotic stress, enzymatic activity was assessed 14 days after infection with fungal pathogens.

**Figure 4:**
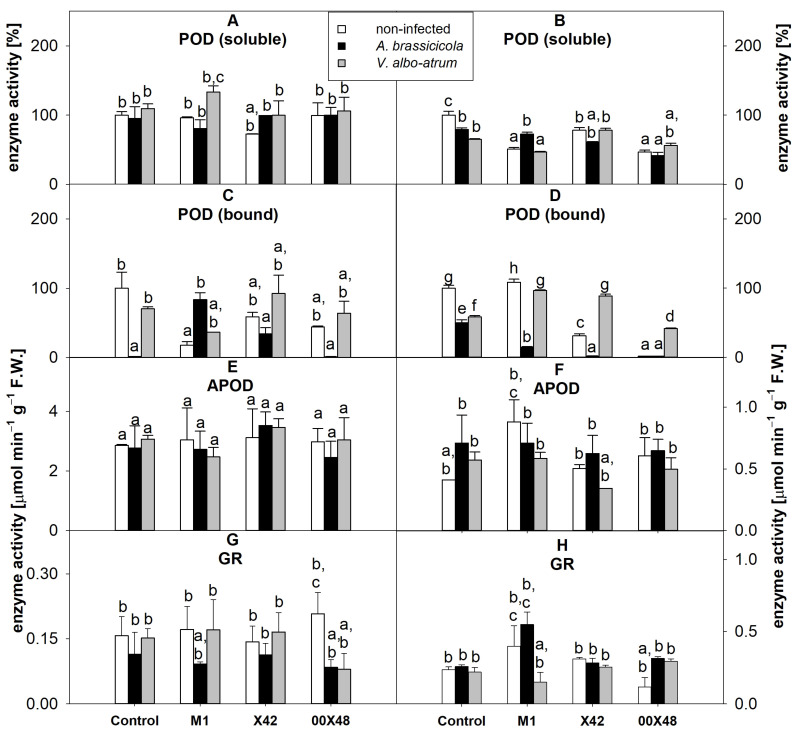
Antioxidant enzyme activity in tomato leaf (**A**,**C**,**E**,**G**) and stem (**B**,**D**,**F**,**H**) extracts from plants grown from seeds treated with a *Pythium* strain (i.e., M1, X42, and 00X48) and from untreated control seeds (Control): soluble peroxidases (**A**,**B**); bound peroxidases (**C**,**D**); ascorbate peroxidase (**E**,**F**); glutathione reductase (**G**,**H**). Plants were infected with *A. brassicicola* (black columns) and *V. albo-atrum* (grey columns) and compared with non-infected control plants (white columns). Different letters above each bar denote significant differences (*p* ≤ 0.05) among plant groups according to one-way ANOVA (Holm–Sidak). The same letters above a bar indicate that no significant differences were found between groups. Each column bar represents the mean ± SD. The degrees of freedom, F-values, and *p*-values can be found in Table S4. APOD, ascorbate peroxidase; F.W., fresh weight; GR, glutathione reductase; PODs, peroxidases.

**Figure 5:**
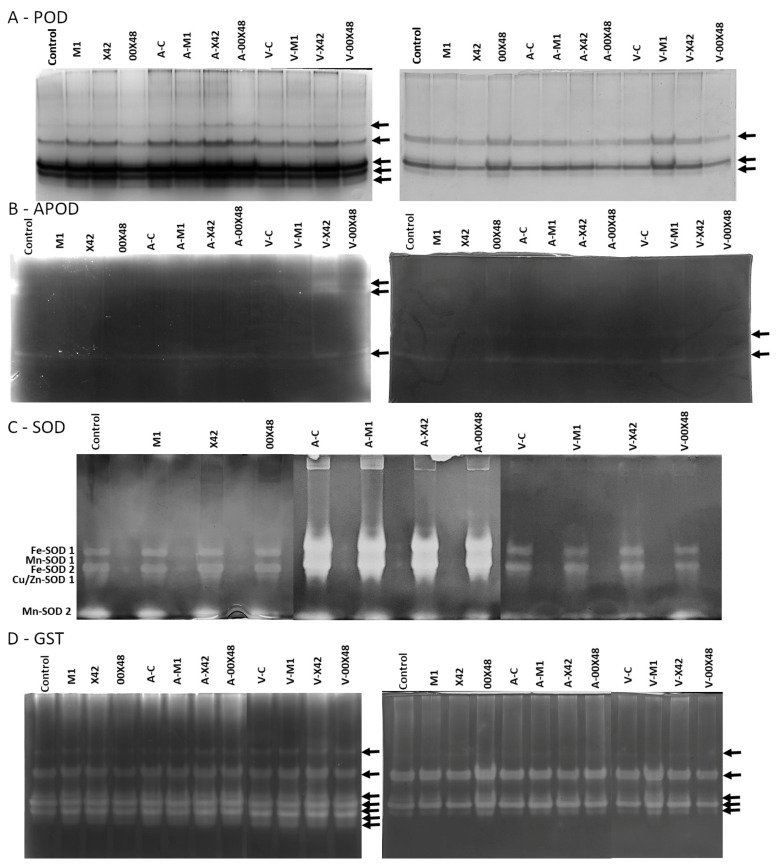
Detection of the activity of peroxidases (**A**); ascorbate peroxidase (**B**); superoxide dismutase (**C**); glutathione-S-transferase (**D**) in leaves (left) and stems (right) after electrophoretic separation under native conditions in 10% polyacrylamide gel. SOD was measured only in leaves. The activity of individual SOD isoenzymes was determined in inhibition studies using H_2_O_2_ and KCN (data not shown). Arrows indicate the main isoforms that are discussed in this study. Abbreviations: A-C, control plants infected with *A. brassicicola*; A-M1, plants treated with M1 and infected with *A. brassicicola*; A-X42, plants treated with X42 and infected with *A. brassicicola*; A-00X48, plants treated with 00X48 and infected with *A. brassicicola*; V-C, control plants infected with *V. albo-atrum*; V-M1, plants treated with M1 and infected with *V. albo-atrum*; V-X42, plants treated with X42 and infected with *V. albo-atrum*; V-00X48, plants treated with 00X48 and infected with *V. albo-atrum*; APOD, ascorbate peroxidase; GST, glutathione-S-transferase; PODs, peroxidases; SOD, superoxide dismutase.

The activity of total soluble peroxidases was not significantly affected by infection, except for leaves from plants infected with *V. albo-atrum* and the seeds of which had been treated with the strain M1. In these leaves, the activity of soluble peroxidases was 1.4 times higher than in leaves from non-infected plants treated with M1 (Figure 4A). In stems, soluble peroxidase activity was slightly lower in all groups than in the untreated control (Figure 4B, Table S3). *A. brassicicola* increased the activity of membrane-bound peroxidases 55 and 23 times in leaves from plants treated with M1 and X42, respectively, in comparison with the untreated infected control (Figure 4C). In stems, the experimental groups infected with *V. albo-atrum* showed higher membrane-bound peroxidase activity than the groups infected with *A. brassicicola*, especially M1 and X42 (Figure 4D). In native electrophoresis, leaf peroxidases were separated into three major bands and a number of minor bands, while only two major bands were visible in stem peroxidases (Figure 5A). In leaves, seed-coating with strain 00X48 led to a less pronounced expression of the middle isoform of peroxidases (second arrow, Figure 5A), while the expression of the last isoform (fifth arrow) was higher in all *Pythium*-treated plants than in the control. In the infected groups, plants infected with both *A. brassicicola* and *V. albo-atrum* and the seeds of which that had been treated with strain X42 showed stronger peroxidase activity in leaves. In stems, the most pronounced bands were identified in the treatment with strain 00X48 and infection with *V. albo-atrum* and treatment with strains M1 and X42 (Figure 5A).

Ascorbate peroxidase (APOD) did not show any significant change in leaves after treatment with *Pythium* strains or infection with pathogens (Figure 4E). In stems, the highest APOD activity was found in the uninfected M1 group (Figure 4F). APOD detection in gel showed a high-mobility band and a weaker low-mobility band. APOD abundance increased after treatment with strain X42 and infection with *V. albo-atrum* in leaves (Figure 5B). The low-mobility band was weaker in stems than in the leaves, but a new band appeared between the two aforementioned bands in leaves.

Glutathione reductase (GR) decreased in all plants infected with *A. brassicicola* and in plants treated with the strain 00X48 and infected with *V. albo-atrum* (Figure 4G). In stems, the GR activity was 2.7 and 2.5 times higher in plants treated with 00X48 and infected with *A. brassicicola* and *V. albo-atrum* than in uninfected 00X48 plants (Figure 4H).

The activity of superoxide dismutase (SOD) was higher in leaves infected with *A. brassicicola* than in those infected with *V. albo-atrum* (Figure 5C). Glutathione-S-transferase (GST), which is involved in detoxification reactions, was also detected by native electrophoresis. Three major bands and a minor band were detected in leaves, but their abundance did not vary with *P. oligandrum* treatment or infection. Two isoforms were clearly visible in stems, with increased activity in plants treated with the strain 00X48 (Figure 5D).

**Figure 6:**
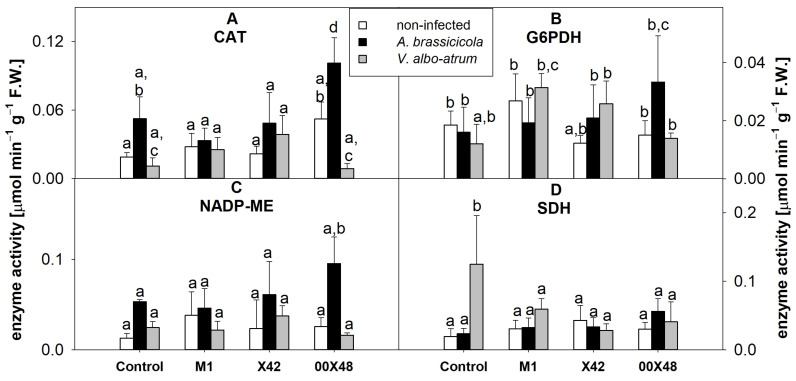
Enzyme activities in tomato leaf extracts from plants grown from seeds treated with a *Pythium* strain (i.e., M1, X42, or 00X48) and from untreated control seeds (Control): CAT (**A**); G6PDH (**B**); NADP-ME (**C**); SDH (**D**). Plants were infected with *A. brassicicola* (black columns) and *V. albo-atrum* (grey columns), and the results were compared with those of non-infected plants (white columns). Different letters above each bar denote significant differences (*p* ≤ 0.05) among plant groups according to one-way ANOVA (Holm–Sidak). The same letters above a bar indicate that no significant differences were found among groups. Each column bar represents the mean ± SD. The degrees of freedom, F-values, and *p*-values can be found in Table S5. Abbreviations: CAT, catalase; F.W., fresh weight; G6PDH, glucose-6-phosphate dehydrogenase; NADP-ME, NADP-malic enzyme; SDH, shikimate dehydrogenase.

Increases in catalase (CAT) activity (i.e., H_2_O_2_ scavenging) are usually related to plant defense, aging, and senescence. CAT activity was 1.8-fold higher in 00X48 leaves infected with *A. brassicicola* than in uninfected 00X48 leaves (Figure 6A). Under various stress conditions, NADPH is supplied by enzyme reactions such as glucose-6-phosphate dehydrogenase (G6PDH) and NADP-malic enzyme (NADP-ME) [33]. G6PDH activity was 1.7- and 2.2-fold higher in leaves from plants infected with *A. brassicicola* and treated with strains X42 and 00X48 if compared to the corresponding uninfected, respectively (Figure 6B). In addition, *V. albo-atrum* infection increased G6PDH activity in leaves from plants treated with strain M1 (1.7-fold) in comparison with uninfected and untreated control leaves (Figure 6B). NADP-ME increased in leaves from all plants treated with *Pythium* strains after infection with *A. brassicicola* in comparison with control leaves (Figure 6C). In particular, the strain 00X48 triggered a 3.7-fold increase in NADP-ME activity. Infection with *V. albo-atrum* also increased NADP-ME activity in the control (1.9 times) and X42 (1.6 times, Figure 6C) samples. Shikimate dehydrogenase (SDH) is a key enzyme of the shikimate pathway, which is closely related to the synthesis of phenolic compounds. Its activity significantly increased after infection with *V. albo-atrum* in the control (6.4 times) and M1 (1.9 times) leaves (Figure 6D).

**Figure 7:**
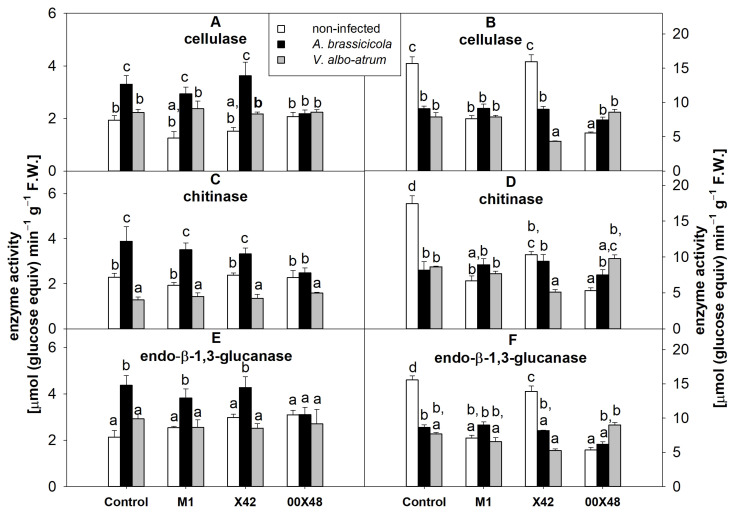
Endoglycosidase activities in tomato leaf (**A**,**C**,**E**) and stem (**B**,**D**,**F**) extracts from plants grown from seeds treated with a *Pythium* strain (i.e., M1, X42, or 00X48) and from untreated control seeds (Control): cellulase (**A**,**B**); chitinase (**C**,**D**); endo-β-1,3-glucanase (**E**,**F**). Plants were infected with *A. brassicicola* (black columns) and *V. albo-atrum* (grey columns), and the results were compared with those of non-infected plants (white columns). Different letters above each bar denote significant differences (*p* ≤ 0.05) among plant groups according to one-way ANOVA (Holm–Sidak). The same letters above a bar indicate that no significant differences were found among the groups. Each column bar represents the mean ± SD. The degrees of freedom, F-values, and *p*-values can be found in Table S6. Abbreviations: F.W., fresh weight.

The activity of soluble endoglycosidases—cellulase, chitinase, and endo-β-1,3-glucanase—was assessed in leaves and stems (Figure 7). In leaves, *A. brassicicola* significantly increased the activity of endoglycosidases in all experimental groups, except for 00X48. After infection with *A. brassicicola*, plants treated with M1 and X42 showed a 2.3- and 2.4-fold increase in cellulase activity and a 1.4- to 1.8-fold increase in chitinase and endo-β-1,3-glucanase activity (Figure 7A,C,E). In turn, the pathogen *V. albo-atrum* caused no significant change in cellulase or endo-β-1,3-glucanase activity but decreased chitinase activity (Figure 7C). The simultaneous effect of *Pythium* and infection (*A. brassicicola* or *V. albo-atrum*) also decreased endoglycosidase activity in control and X42 stems (Figure 7B,D,F). Table 2 summarizes statistical differences of results as determined by one-way ANOVA. 

**Figure 8:**
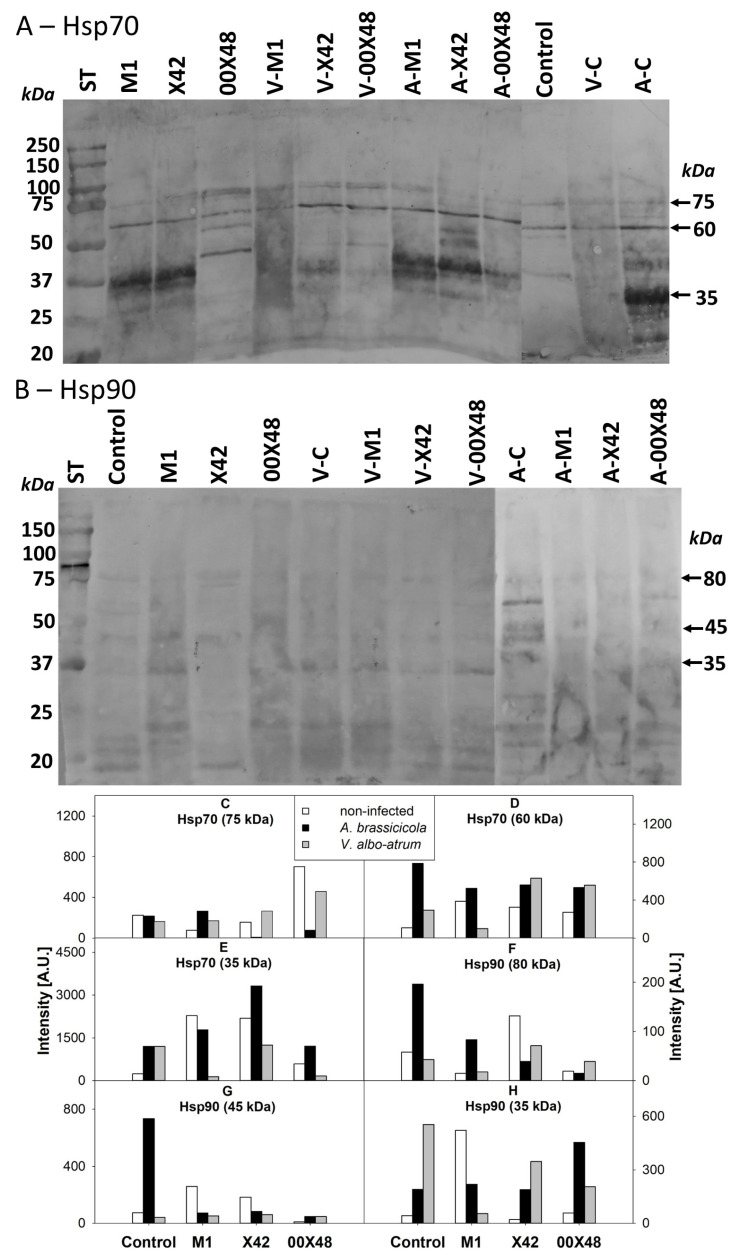
Relative content of cytosolic Hsp70 (**A**) and Hsp90 (**B**) determined by immunoblotting after SDS-electrophoresis in tomato leaf extracts from plants grown from seeds treated with a *Pythium* strain (i.e., M1, X42, or 00X48) and from untreated control seeds (**Control**). The relative intensity of bands (**C**–**H**) was evaluated in GelAnalyzer 19.1. Abbreviations: A-C, control plants infected with *A. brassicicola*; A-M1, plants treated with M1 and infected with *A. brassicicola*; A-X42, plants treated with X42 and infected with *A. brassicicola*; A-00X48, plants treated with 00X48 and infected with *A. brassicicola*; V-C, control plants infected with *V. albo-atrum*; V-M1, plants treated with M1 and infected with *V. albo-atrum*; V-X42, plants treated with X42 and infected with *V. albo-atrum*; V-00X48, plants treated with 00X48 and infected with *V. albo-atrum*; ST, Precision Plus Protein™ Kaleidoscope™ by Bio-Rad. Arrows point at proteins discussed in the text.

Leaf Hsp70 immunochemical detection on nitrocellulose membranes showed the three most visible isoforms, with molecular weights of 75, 60, and 35 kDa. *A. brassicicola* had a stronger effect on the abundance of Hsp70 isoforms than *V. albo-atrum*. For example, in non-treated plants, the 60-kDa isoform was also more abundant in plants infected with *A. brassicicola*. Some isoforms were also decreased by fungal infection (Figure 8A). Similarly, the three main Hsp90 isoforms of 80, 45, and 35 kDa were followed, and the results showed that, after infection, the levels of most isoforms were lower in leaves from plants treated with M1 and X42 than in infected control leaves. Conversely, the intensity of all Hsp90 isoforms increased in leaves of untreated plants infected with *A. brassicicola* (Figure 8B).

## 4. Discussion

The oomycete *P. oligandrum* is able to induce defense responses in plants and develop resistance to fungal, oomycete, and bacterial pathogens. The extent of protection depends on many parameters including the method of application, i.e., spraying, watering, or seed treatment [5]. In our previous study, we showed that seed-coating leads to a number of metabolic changes in rapeseed plants including free amino acids, phytohormones, and glucosinolates; significant differences were also found among *P. oligandrum* strains such as secretion of enzymes and elicitors [24]. Here, we focused on determining if seed treatment with *Pythium* strains also affected the metabolism of tomato plants and induced defense responses against the fungal pathogens *A. brassicicola* and *V. albo-atrum*. For this research, we chose the most advantageous strains from our previous study, i.e., M1, X42, and 00X48 [24].

All *Pythium* strains tested in this study successfully inhibited *A. brassicicola* and *V. albo-atrum* growth in vitro (Figure 1 and Figure 2). Mycoparasitism manifested as *Pythium* hyphae coiling around the pathogen hyphae and pathogen hyphae growth inhibition (Figure 1). *Pythium* strain X42 showed the highest inhibition effect on both fungal pathogens, with minimal differences in inhibition percentage nevertheless (Figure 2). Additionally, *P. oligandrum* strain M1 produced a large number of aerial hyphae, thus significantly overgrowing *V. albo-atrum*. Together with high glycosidase and protease activities [24], *Pythium* strains show advantageous properties for biological control.

Plants treated with *Pythium* strains showed a significantly higher content and distribution of free amino acids than the untreated control plants (Figure 3, Table 1). However, their protein content remained unchanged. Considering the effect of *P. oligandrum* on plants, the amino acid tryptophan stands out because *P. oligandrum* exchanges tryptamine, a phytohormone auxin precursor, for plant tryptophan [5]. In this study, the tryptophan content was significantly lower in tomato leaves from plants treated with the strain 00X48 and in all infected groups than in the control group (Table 1). This decrease in the tryptophan content of infected plants may also be related to the production of tryptophan-derived secondary metabolites, which likely contribute to defense mechanisms against fungal pathogens, as shown for *V. longisporum* in *Arabidopsis thaliana* roots [34]. During the *in planta* experiments, we aimed to analyze the changes in plant antioxidant system at the early stages of fungal infection. Because seed-coating could be especially helpful in the beginning of plant growth and development. Not many significant differences were found in the plant antioxidant system, likely because the infection had not fully infested the plant. At the tested experimental stage of infection, the first metabolites to respond and change their concentration were free amino acids and heat shock proteins, and the activities of SOD and endoglycosidases also increased in the case of *A. brassicicola*.

The metabolic changes in tomato plants caused by *Pythium*-treated seeds can be compared with those in rapeseed plants from our previous study [24]. Differences between treated and untreated plants were greater in the tested parameters in the case of rapeseed plants. Thus, the plant response to seed-coating with *Pythium* strains seems to be species specific. There is a significant difference between tomato and rapeseed plants in regard to the distribution of the individual free amino acids. But changes in tryptamine content, which *P. oligandrum* can provide to the plant for auxin synthesis, were small compared to rapeseed plants. The effect of *P. oligandrum* strains on the activity of antioxidant enzymes in tomato and rapeseed plants was similar. While the activity of CAT and GR was not affected, the activity of some SOD isoforms increased (Figure 4G, Figure 5C and Figure 6A). In addition, the total phenolic content and antioxidant capacity did not differ between the control and treated tomato plants, and rapeseed plants gave similar results. An explanation for this may be the incorporation of phenolic compounds into the cell wall and, thus, its strengthening. In this context, an increase in the activity of peroxidases or in the number of isoforms may provide plants with an advantage in their defense against fungal pathogens either through the formation of reactive oxygen species or through cell wall lignification. Seed-coating with *Pythium* strains affected the peroxidase isoform content, because the isoform with the highest mobility (fifth arrow, Figure 5A) had a stronger activity band in the corresponding plants than in untreated control plants in this study. APOD also showed significantly stronger activity bands with low mobility in leaves from plants treated with X42 and 00X48 and infected with *V. albo-atrum* (Figure 5B).

Although both *A. brassicicola* and *V. albo-atrum* were detected by qRT-PCR in tomato leaves (Figure 3A), *V. albo-atrum* levels were lower in tomato plants treated with the strains 00X48 and X42. However, at this early stage of infection, it points more to the pathogen’s presence than disease development. Together with other experimental results, we would suggest combining *Pythium* strain seed-coating with other application methods to ensure effective long-term plant protection.

The content of free amino acids in control plants decreased upon fungal infection. By contrast, after treatment with *Pythium* strains, this content either increased or remained unchanged upon infection. Amino acid homeostasis is crucial for plant growth, development, and defense, and it is affected by amino acid synthesis, uptake, and transport and by protein synthesis and degradation [35]. In fungus-infected leaves, the increase in amino acids could reflect an increase in apoplastic protease activity induced by infection. Fungal pathogens may either manipulate plant metabolism to maintain or increase the apoplastic concentration of nitrogen compounds for their own use [36]. Amino acids can also interconvert, depending on the actual conditions and the course of infection.

Glutathione plays a key role in plant defense reactions; a concentration of cysteine below the limit of detection as a reactive compound likely indicates its incorporation into the glutathione molecule. Glycine, another component of glutathione, is also reduced in most infected plants (Table 1). However, as the simplest amino acid, glycine is involved in the synthesis of other molecules, such as phospholipids. Glutamate, another component of glutathione, is one of the basic amino acids that is used not only in transamination reactions but also in the synthesis of other substances including the amino acids proline and arginine, chlorophyll, and cytochromes [37]. During sunflower infection with the necrotrophic fungus *Botrytis cinerea*, glutamate may be transferred to the invaded region as a nitrogen source to delay cell death and disturb fungal progression in plant tissues [36]. In plants treated with the strains M1 and 00X48, glutamate increased after infection with *V. albo-atrum* but decreased after infection with *A. brassicicola* (Table 1). Because our experiments were based on mixed leaf samples, we were unable to follow glutamate distribution between regions. Nevertheless, significant glutamate increases may be related to activated defense reactions in invaded areas.

Plant defense pathways are regulated not only by phytohormones but also by distinct amino acid metabolic pathways, which are integral to the plant immune system, with aspartate oxidation and subsequent pyridine nucleotide formation emerging as an important metabolic route for pre- and post-invasion plant defense [38]. Accordingly, aspartate was increased in all pathogen-infected experimental groups (Table 1). Branched-chain amino acid (Leu, Ile, and Val) catabolism may also affect crosstalk between the phytohormones salicylic acid (SA) and jasmonate (JA), thereby affecting plant disease resistance [38]. Corroborating these findings, Leu, Ile, and Val showed similar increases and decreases in fungal-treated plants in comparison with untreated healthy controls (Table 1).

Crosstalk between other amino acids may be involved in the synthesis and metabolism of phytohormones. For example, JA and ethylene (ET) are important for defense reactions against fungal diseases. In line with the above, methionine, which is a precursor of ET, showed a two-fold increase in plants treated with the strains X42 and 00X48 and infected with *V. albo-atrum*, where pathogen abundance was reduced. JA was active only after conjugation with isoleucine, the plant groups protected against *V. albo-atrum* (i.e., X42 and 00X48) did not have a reduced amount of isoleucine, in contrast to the other infected plant groups (Table 1, Figure 3A), suggesting defense response. In our previous study, we reported that the higher content of phytohormones JA, as well as SA, was related to plant response to *Pythium* seed treatment of rapeseed plants [24].

In general, plants defend themselves against fungal diseases by increasing the activity of glycosidases, namely, cellulases, chitinases, and glucanases, which can degrade cell wall components of pathogens. These enzymes are also referred to as PR proteins and are synthesized by plants in response to biotic stress [39]. Endoglycosidases are also secreted by *P. oligandrum*, and they are important for its mycoparasitism [24]. In this study, tomato seed-coating alone with *Pythium* strains had no significant effect on the activity of these enzymes in leaves (Figure 7) even though oligandrin application to tomato leaves is known to up- or downregulate the expression of β-1,4-glucanase, chitinase, and β-1,3-glucanase genes [40]. In the leaves of *A. brassicicola*-infected plants, the highest activities of cellulase, chitinase, and endo-β-1,3-glucanase were found (Figure 7A,C,E). A possible explanation for these results may lie in local changes in the activities of these enzymes; in this study, we worked with mixed samples, which mask these local changes. Nevertheless, *A. brassicicola* infection was more severe and had a stronger effect on tomato metabolism than *V. albo-atrum* infection.

Hsps, originally identified as proteins strongly increased by heat treatment, are crucial for plant growth and perform a range of other functions including plant defense [41,42]. The critical role of cytosolic Hsp70s in plant defense is supported by the fact that these proteins are targeted by pathogen effector proteins. For example, transient Hsp70 overexpression in *Nicotiana benthamiana* inhibits *Phytophthora* growth [41]. In tomato plants, 23 genes encode Hsp70 proteins, namely, 3 Hsp70 proteins with 92–98 kDa, 2 with 77–80 kDa, 14 with 70–75 kDa, 3 with 62–67 kDa, and 1 with 21 kDa [22]. In our analysis, the increase in Hsp70s (50–70 kDa, Figure 8A) in seed-coated plants may partly account for the priming effect of the interaction between the plant and *P. oligandrum.* The initial higher content of Hsp70s may help the plant respond to fungal pathogens more efficiently. In previous studies, Hsp70 has been shown to be required for resistance to *Pseudomonas chicorii* in tobacco plants and to mediate Arabidopsis defense against bacterial and oomycete pathogens [42]. In such effector-triggered immunity, the Hsp70 interactor SGT1 plays a key role, in addition to regulating auxin and JA signaling by maintaining steady-state levels of the corresponding receptors. Moreover, SGT1 is also a co-chaperone of Hsp90 [41].

Such Hsp90-associated chaperoning is important for maintaining immunity activity triggered by pathogen-associated molecular patterns. In fact, tomato plants contain at least seven Hsp90 genes with protein lengths ranging from 267 to 794 amino acids. Hsp90s are not only involved in responses to abiotic stress but also in resistance to pathogens [23]. In our analysis (Figure 8B), we identified Hsp90.1, Hsp90.5, Hsp90.7 (80.27, 80.14, and 80.16 kDa), and Hsp90.6 (31.30 kDa). The bands of approximately 45–60 kDa may correspond to degradation products or co-chaperones. Therefore, our results are in line with previous findings, which showed that Hsp90 is particularly relevant in tomato plant resistance against infection with *Tomato Yellow Leaf Curl Virus* and tobacco against *Potato virus Y* [43,44].

## 5. Conclusions

In vitro dual culture tests clearly demonstrated the mycoparasitic ability of all three *Pythium* strains (i.e., M1, X42, and 00X48) against fungal pathogens *A. brassicicola* and *V. albo-atrum*. Seed-coating with *Pythium* strains is an environmentally friendly approach to protecting tomato plants. Although no visible differences were detected among the four-week-old plants, the free amino acid content and activities of some antioxidant enzymes changed. After seed treatment with *Pythium* strains, pathogen content only decreased in plants infected with *V. albo-atrum*. This outcome necessarily depends on the environmental conditions, the plant species and its cultivar, and on the type of pathogen. Nevertheless, these results show that seed treatment with *Pythium* strains can help plants germinate, especially in the presence of pathogens, but at later stages of development, this treatment should be supplemented with further applications, either by watering or spraying. In future research for a more conclusive decision as to whether the new *Pythium* strains X42 and 00X48 are suitable as biological control agents, these experiments should be extended to the entire course of the infections, and seed-coating should be combined with an application of *Pythium* strains to the soil. Furthermore, combining *Pythium* strains with each other or with other beneficial microorganisms could lead to a more effective and complex plant protection.

## Figures and Tables

**Table 1:** Content of individual free amino acids of tomato leaf extracts from plants grown from seeds treated with a *Pythium* strain (i.e., M1, X42, and 00X48) and infected with *A. brassicicola* or *V. albo-atrum*. The content of individual amino acids of all samples is expressed as the % of the total amount of amino acids and compared with the corresponding value of the control (C, untreated and non-infected plants). The blue color highlights higher values than the control, and the red color highlights the values lower than the control. Asterisks denote significant differences (*p* ≤ 0.05) between sample and control plants according to a *t*-test. Abbreviations: A-C, control plants infected with *A. brassicicola*; A-M1, plants treated with M1 and infected with *A. brassicicola*; A-X42, plants treated with X42 and infected with *A. brassicicola*; A-00X48, plants treated with 00X48 and infected with *A. brassicicola*; V-C, control plants infected with *V. albo-atrum*; V-M1, plants treated with M1 and infected with *V. albo-atrum*; V-X42, plants treated with X42 and infected with *V. albo-atrum*; V-00X48, plants treated with 00X48 and infected with *V. albo-atrum*; <LOD, under the limit of detection.

	C	M1	X42	00X48	A-C	A-M1	A-X42	A-00X48	V-C	V-M1	V-X42	V-00X48
**Alanine**	100 ± 5%	114 ± 4%	131 ± 17%	112 ± 4%	94 ± 5%	105 ± 7%	127% ± 1 *	101 ± 1%	80 ± 19%	137 ± 15%	145 ± 9% *	119 ± 13%
**Arginine**	100 ± 6%	87 ± 9%	132 ± 16%	78 ± 2%	84 ± 6%	117 ± 12%	108 ± 1%	96 ± 11%	29 ± 2% *	69 ± 27%	124 ± 9%	118 ± 2%
**Asparagine**	100 ± 13%	113 ± 7%	74 ± 5%	187 ± 6%	36 ± 3% *	62 ± 6%	73 ± 6%	127 ± 2% *	57 ± 1% *	45 ± 3% *	69 ± 7%	53 ± 5%
**Aspartate**	100 ± 0%	108 ± 9%	74 ± 16%	100 ± 6%	188 ± 17% *	177 ± 12% *	132 ± 9% *	124 ± 5% *	129 ± 14%	149 ± 32%	125 ± 18%	124 ± 10% *
**Cysteine**	<LOD	<LOD	<LOD	<LOD	<LOD	<LOD	<LOD	<LOD	<LOD	<LOD	<LOD	<LOD
**Glutamine**	100 ± 5%	107 ± 6%	72 ± 11%	156 ± 5%	60 ± 6% *	95 ± 6%	72 ± 3% *	127 ± 0% *	59 ± 6% *	80 ± 14%	78 ± 6% *	63 ± 4% *
**Glutamate**	100 ± 1%	4 ± 0% *	48 ± 4% *	67 ± 5% *	168 ± 16% *	6 ± 0% *	19 ± 8% *	43 ± 3% *	331 ± 34% *	163 ± 26%	8 ± 1% *	174 ± 5% *
**Glycine**	100 ± 4%	45 ± 16%	165 ± 30%	57 ± 7%	25 ± 10% *	55 ± 4% *	104 ± 9%	72 ± 11%	38 ± 17% *	65 ± 35%	73 ± 3% *	98 ± 21%
**Histidine**	100 ± 8%	100 ± 6%	114 ± 16%	58 ± 3%	31 ± 3% *	58 ± 8% *	86 ± 10%	65 ± 6% *	25 ± 0% *	54 ± 16%	75 ± 10%	66 ± 7%
**Isoleucine**	100 ± 2%	81 ± 7%	128 ± 17%	67 ± 6%	59 ± 5% *	70 ± 4% *	99 ± 3%	74 ± 9%	49 ± 13% *	76 ± 14%	102 ± 11%	97 ± 11%
**Leucine**	100 ± 2%	88 ± 2% *	134 ± 4% *	70 ± 3% *	71 ± 12%	79 ± 5% *	112 ± 4%	80 ± 2% *	45 ± 8% *	84 ± 10%	116 ± 6%	109 ± 3%
**Lysine**	100 ± 1%	94 ± 8%	147 ± 16%	92 ± 2%	86 ± 8%	89 ± 8%	130 ± 3% *	96 ± 2%	59 ± 7% *	87 ± 25%	117 ± 7%	119 ± 5% *
**Methionine**	100 ± 4%	199 ± 30%	555 ± 91% *	45 ± 5% *	<LOD *	69 ± 4% *	63 ± 5% *	111 ± 16%	126 ± 14%	0 ± 0% *	215 ± 59%	230 ± 20% *
**Phenylalanine**	100 ± 9%	103 ± 5%	105 ± 14%	90 ± 6%	70 ± 11%	92 ± 5%	104 ± 11%	111 ± 3%	338 ± 49% *	104 ± 14%	103 ± 15%	101 ± 9%
**Proline**	100 ± 11%	154 ± 15%	158 ± 28%	49 ± 4%	50 ± 4% *	79 ± 14%	110 ± 11%	92 ± 6%	33 ± 2% *	59 ± 2%	97 ± 13%	72 ± 14%
**Serine**	100 ± 2%	90 ± 10%	100 ± 13%	90 ± 0%	75 ± 15%	71 ± 2% *	82 ± 4% *	77 ± 3% *	52 ± 10% *	58 ± 10% *	79 ± 4% *	88 ± 8%
**Threonine**	100 ± 2%	111 ± 6%	97 ± 8%	94 ± 3%	96 ± 14%	97 ± 2%	101 ± 6%	90 ± 3%	86 ± 1% *	86 ± 27%	128 ± 9%	99 ± 2%
**Tryptophan**	100 ± 3%	104 ± 9%	106 ± 6%	24 ± 0%	64 ± 3% *	36 ± 6% *	69 ± 3% *	41 ± 5% *	39 ± 1% *	63 ± 25%	48 ± 6% *	41 ± 4% *
**Tyrosine**	100 ± 5%	99 ± 5%	134 ± 0%	83 ± 9%	105 ± 10%	85 ± 11%	95 ± 4%	90 ± 6%	47 ± 4% *	103 ± 17%	91 ± 11%	85 ± 17%
**Valine**	100 ± 2%	107 ± 4%	133 ± 28%	83 ± 5%	83 ± 7%	93 ± 3%	115 ± 4% *	84 ± 3% *	71 ± 18%	90 ± 5%	132 ± 11%	118 ± 7%

**Table 2:** Statistical analysis: data were analyzed according to one-way ANOVA (Holm–Sidak) in SigmaPlot 12.5. Abbreviations: DF, degree of freedom.

Figure	Determination	DF between Groups	DF—Residual	F-Value	*p*-Value
in vitro test	*Alternaria brassicicola*	2	8	9.431	0.014
	*Verticillium albo-atrum*	2	8	0.362	0.711
Figure 3	pathogen content	7	16	4.23	0.008
	protein content	11	46	1.076	0.4
	total aa content	11	24	25.589	<0.001
	tryptamine content	11	24	5.518	<0.001
	total phenolics	11	36	3.851	0.001
	antioxidant capacity	11	36	2.447	0.021
Figure 4	peroxidases/POD soluble—leaves	11	24	4.138	0.002
	peroxidases/POD bound—leaves	11	24	22.586	<0.001
	peroxidases/POD soluble—stems	11	24	93.534	<0.001
	peroxidases/POD bound—stems	11	24	861.548	<0.001
	ascorbate peroxidase/APOD—leaves	11	24	0.886	0.566
	ascorbate peroxidase/APOD—stems	11	18	3.883	0.005
	glutathione reductase/GR—leaves	11	39	3.456	0.002
	glutathione reductase/GR—stems	11	12	7.614	<0.001
Figure 6	catalase/CAT	11	46	14.398	<0.001
	glucose-6-phosphate dehydrogenase/G6PDH	11	36	3.725	0.001
	malic enzyme/NADP-ME	11	37	5.636	<0.001
	shikimate dehydrogenase/SDH	11	47	5.94	<0.001
Figure 7	cellulase—leaves	11	24	23.694	<0.001
	chitinase—leaves	11	24	32.412	<0.001
	endo-β-1,3-glucanase—leaves	11	24	13.506	<0.001
	cellulase—stems	11	24	115.718	<0.001
	chitinase—stems	11	24	60.431	<0.001
	endo-β-1,3-glucanase—stems	11	24	154.071	<0.001

## Data Availability

Not applicable.

## Supplementary Materials

The following supporting information can be downloaded at: https://www.mdpi.com/article/10.3390/microorganisms10071348/s1, Tables S1–S6: Statistical analyses.
